# Haze smoke impacts survival and development of butterflies

**DOI:** 10.1038/s41598-018-34043-0

**Published:** 2018-10-23

**Authors:** Yue Qian Tan, Emilie Dion, Antónia Monteiro

**Affiliations:** 1 Department of Biological Sciences, National University of Singapore, 14 Science Drive 4, Singapore, 117543 Singapore; 2 Yale-NUS-College, 6 College Avenue East, Singapore, 138614 Singapore

## Abstract

The Southeast Asian transboundary haze contains a mixture of gases and particles from forest fires and negatively impacts people’s health and local economies. However, the effect of the haze on organisms other than humans has not yet been sufficiently studied. Insects are important members of food webs and environmental disturbances that affect insects may impact whole ecosystems. Here we studied how haze directly and indirectly affects the survival, growth, and development of insects by rearing *Bicyclus anynana* butterflies under artificially generated smoke as well as reared in clean air but fed on plants previously exposed to smoke. Direct haze exposure significantly increased the mortality of caterpillars, increased larval development time, and decreased pupal weight, while indirect haze exposure, via ingestion of haze-exposed food plants, also affected development time and pupal weight. No smoke particles were found in the tracheae of subjects from the smoke treatment suggesting that the increase in development time and mortality of *B. anynana* under smoke conditions might be due to toxic smoke gases and toxic food, rather than particulate matter. These results document significant deleterious effect of haze smoke to the development, adult size, and survival of insects, key players in food-webs.

## Introduction

The Southeast Asian (SEA) transboundary smoke plumes, commonly known as “haze”, occur when commercial tree plantation developers and local farmers, mainly from the Indonesian islands of Sumatra and Borneo, clear forest lands using fire^1,2^. The phenomenon peaks during the intermonsoon dry season between June to October and is exacerbated when there are prolonged draughts during El Nino years^1,3^. When carried by the monsoon winds, the haze affects local dwellers as well as those in neighboring countries such as Malaysia and Singapore^4–7^.

The haze impacts both human health and local economies^8,9^. Field studies have identified over 100 compounds in the smoke from forest fires. They include carbon dioxide and monoxide (CO_2_ and CO), methane (CH_4_), nitrogen oxides (NOX), sulphur dioxide (SO_2_), polycyclic aromatic hydrocarbons (PAHs), and smoke particles (also known as particulate matter, PM)^10^. Particulate matter less than 2.5 μm in aerodynamic diameter (PM2.5), especially, can invade the smallest airways in the lungs, aggravating respiratory and cardiovascular diseases^11–13^. For instance, the 1997 SEA haze episode reached hazardous levels of ground-level particulate matter concentrations, and was followed by an increase in respiratory problems in Singapore, together with a 2% annual increase in regional adult cardiovascular mortality^12^. In addition, haze has brought economical losses because of falls in tourism, aviation, industrial investment and production^6^. Despite the high number of studies on the effect of the haze on human health and lifespan, its impact on other species and on ecosystems has rarely been addressed.

Herbivorous insects are primary consumers that link plants to higher-level consumers in food webs. They are also key players in many ecological functions^14^, such as in plant pollination^15^, seed dispersal^16,17^, or soil aeration^18^. Because some insects have high sensitivity to environmental change, they are used as bioindicators of the health of a habitat or an ecosystem (e.g.^19,20^). Butterflies, in particular, are used for these purposes because, in addition to their sensitivity to environmental disturbances, they are easy to identify and monitor. In Indonesian forests, for instance, butterfly species richness and abundance was directly linked to human activities such as forest logging and fires, presumably via direct destruction of their habitat^21,22^. In addition to direct impacts, forest fires can have indirect effects on butterflies as well as other insects via the generation of smoke plumes. No study, however, has investigated the effects of smoke haze on insects.

One way in which haze might affect insects is by impacting their respiratory system, as it does for humans. Insects breath air via spiracles, valve-like openings on the side of their bodies that connect to internal tracheal tubes^23,24^ which branch repeatedly into finer tracheoles that eventually reach every cell inside the insect’s body, where diffusion of gases occurs^25^. Insects such as flies, beetles, crickets or moths, among others, can also use an active respiration process where rapid cycles of compression and expansion of the trachea and the air sacs in various parts of the body create air movement^26–30^. An insect’s aeration system constitutes, thus, a direct path to all internal organs making it extremely vulnerable to air quality. For instance, insecticide injected into spiracles leads to immediate and severe paralysis compared to a topical cuticular application^31^.

This study aims to evaluate the effects of haze smoke on insect larval growth and survival to understand how insects might respond to SEA haze episodes. We used the nymphalid butterfly *Bicyclus anynana* as a test species. We hypothesized that haze has a detrimental effect on fitness, either via mechanical obstruction and damage of the fine tracheoles by the particular matters, via gas toxicity, or via food plant toxicity, when food plants alone are exposed to haze. In two sets of experiments, we exposed individuals to smoke throughout their entire development, from egg to adulthood, and in another set we exposed food plants to haze and fed individuals on these exposed plants for their entire larval development, from 1^st^ instar to pre-pupae. We measured development time, weight at pupation, and mortality. We also performed a histological examination of the larvae trachea, and analyzed the structure of the larval spiracles via scanning electron micrographs, to gain extra insights into the mode of action of smoke on insect mortality.

## Methods

### Subjects and experimental setup

Eggs of *Bicyclus anynana* were collected from a laboratory stock reared in a climate room at 27 °C and 60% relative humidity. On the fifth day after hatching, larvae were sorted into treatment groups. The experiments were carried out outdoors, under an open-air shed, protected from direct sunlight.

**Figure 1:**
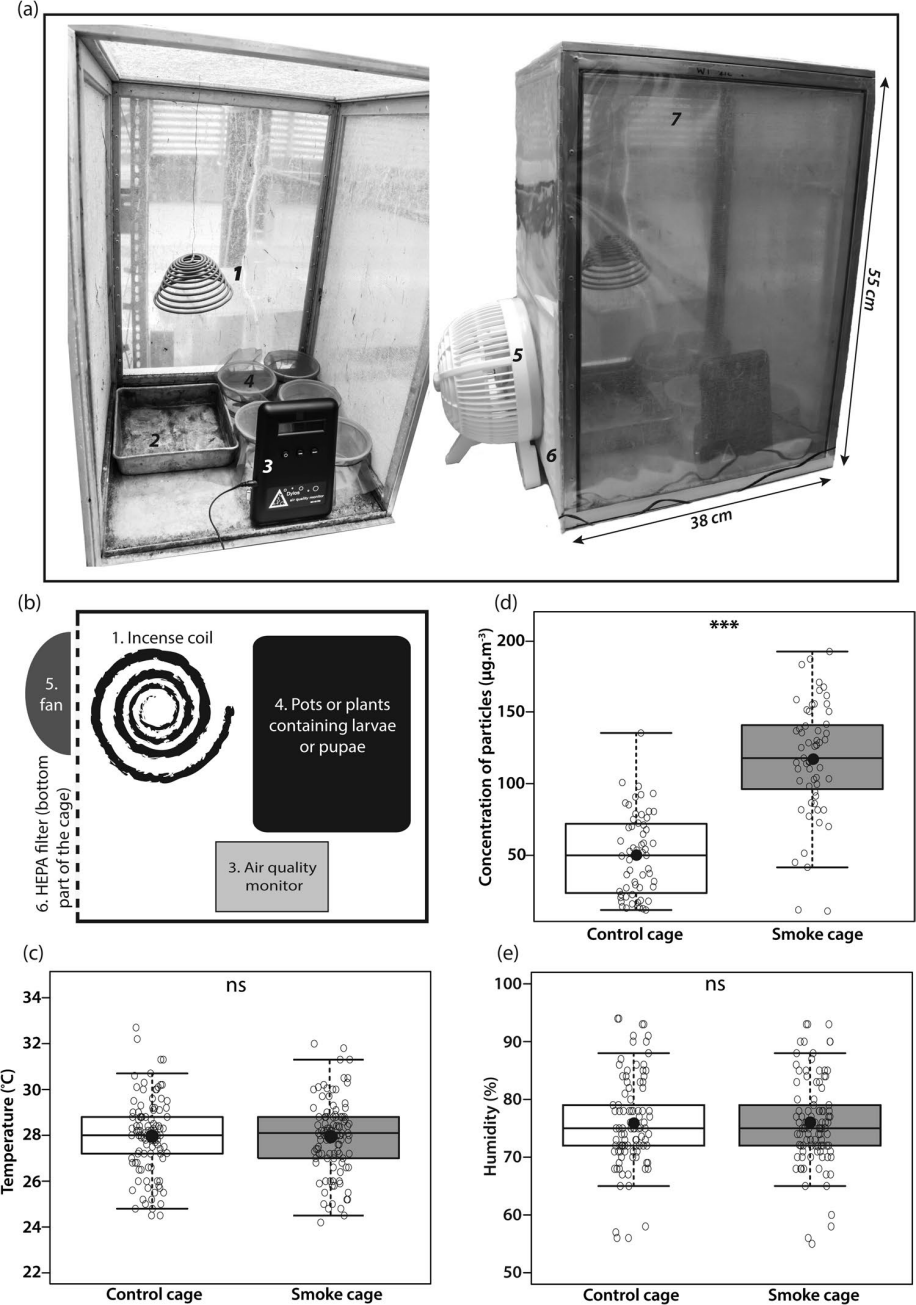
Experimental set-up and environmental conditions inside the cages. (**a**,**b**) Individuals were grown in plastic containers or on plants (4), inside cages covered with transparent PVC sheets (7). Each smoke cage contained an incense coil (1), a metal container collecting the ashes from the coil (2), and an air quality monitor (3). A fan (5) was placed facing the bottom part of the cage on one side covered by an HEPA filter (6). The control cages’ set-up was identical, except for the incense coil and the metal container that were removed. (**c**,**d**) Temperature and humidity were similar in both control and smoke cages. (**d**) Particle concentration was significantly higher in the smoke cage than in the control cage. In panels c, d and e, the higher and lower bars of the plot are the maximum and minimum values respectively, while the rectangle illustrates the first quartile, the median, and the third quartile (bottom to top). The black dot is the average, and each open circle is a data point. Significant differences between treatments are represented by asterisks: *p < 0.05; **p < 0.01, ***p < 0.001, ns = non-significant.

The experiments were conducted inside wire mesh cages which sides were sealed by transparent PVC sheets. The bottom half of one of the sides was covered with a High Efficiency Particulate Air (HEPA) filter. To facilitate air flow inside the cage, a fan was placed outside facing the filter (Fig. 1a,b). We simulated haze using incense smoke, because of their similar composition and the incense’s ability to sustain combustion over time (one coil lasted around 24 hours). Incense is generally made of a paste of rounded wood matter along with organic adhesive and potassium nitrate to facilitate combustion. Pollutants emitted from burning incense include CO, NO, SO_2_, carbonyls, volatile organic compounds, PAHs, PM10 (particulate matter less than 10 µm in aerodynamic diameter), and PM2.5^32–34^. In one of the cages from each treatment type, a DC1100 Pro air quality monitor (Dylos Corporation) was placed to log the daily average of smoke particle concentration (Fig. 1a,b). Temperature and humidity in the 4 cages were recorded once every day around 5 pm with a digital thermohygrometer to ensure that these conditions were constant across both treatments (Fig. 1c–e).

### Direct effect of smoke on *B. anynana* development

We conducted two experiments (experiments 1 and 2) that differed in how the larvae were fed. In experiment 1, two cages were employed for each treatment group, Smoke and Control, making a total of four cages. Larvae were placed in groups of ten into cylindrical plastic containers, and five containers were used in each cage. Larvae were given ~0.075 g/per individual of pieces of fresh young corn leaves every day. We recorded the number of surviving larvae in each container on a daily basis. In addition, we recorded the time taken by each larva to reach pupation, the number of pupae that successfully emerged, and the total mortality from egg to emergence.

In experiment 2, one cage per treatment was used. Caterpillars were reared on potted corn plants changed every one to two days, with ad-libitum access to food. Larvae were placed in groups of ten per plant inside a separate mesh container, and five plants/containers were used in each cage. In addition to the same parameters described above, we also recorded the time taken by each pupae to emerge as a butterfly; the total development time from egg to adult; and the mass of each pupa on the day of pupation. Individuals that perished during the experiment were collected and kept at −20 °C for subsequent analyses.

### Indirect effect of smoke-induced plant toxicity on *B. anynana* development

In experiment 3, we fed newly hatched caterpillars with 2-week-old potted corn plants that were either previously placed in the haze environment or in the control environment for 24 hours. As in experiment 2, the larvae were placed in groups of ten per plant inside mesh containers, with five plants used from each environment. All larvae from both treatments were kept in a control cage environment. Similar to experiment 2, we measured the time taken by each larva to reach pupation and emergence, the larval, pupal and total mortality, and the pupal mass on the day of pupation.

### Histological examination of the tracheal system

We investigated the presence of smoke micro-particles in the insects’ tracheal system using light microscopy. Trachea sections from the thoracic segments (air intake trachea) were dissected in PBS and transferred to 1% glutaraldehyde in 0.1 M phosphate buffer, pH 7, for 5 minutes. Samples were then mounted onto glass slides, and observed using a Zeiss Axio Imager M2 microscope.

### Spiracle structure examination using scanning electron microscopy (SEM)

To investigate for the potential presence of micro particles at the spiracle openings, larval specimens were dried in an incubator at 37 °C until constant dry mass, then sputtered with a thin layer of gold using JEOL JFC 1100 ions sputtering machine, and imaged under a JEOL JSM-6510LV SEM.

### Statistical analyses

Since we used only one or two cages per treatment (where each cage contained multiple groups of larvae), our results may suffer from a pseudoreplication problem (but see^35^). We accounted for this by using mixed models, which is a statistical procedure commonly used to correct for pseudoreplication^36,37^.

The effect of the smoke on the temperature, humidity and PM2.5 concentrations were analyzed in two steps. (1) since [PM2.5] was measured in only one cage of each treatment, whereas temperature and humidity were measured in 2 cages each, we analyzed the effect of the cage on these two factors separately by fitting a linear mixed model (LMM), where treatment (smoke or control) was a fixed factor, and cage (two different cages per treatment) a random factor^37^. The effect of the cage factor on humidity and temperature was tested using Likelihood Ratio Tests (LRTs) of full models tested against simplified models with the specific factor removed. (2) As temperature, humidity and [PM2.5] are dependent factors, we also applied a Multivariate Analysis of Variance (MANOVA) using a Pillai test, followed by univariate ANOVA on each variable. Since temperature and humidity were the same across the four cages (see results), we used the values from one cage of each treatment to perform the MANOVA. Multivariate normality was checked using Mardia’s tests, and univariate normality and homoscedasticity tested using Shapiro-Wilk and Levene’s tests.

A LMM was used to analyze the effect of smoke on the average time taken by caterpillars to reach pupation, the average time taken by pupae to emerge as butterflies, the average total development time, and the average pupal weight, with the plastic container or group of caterpillars reared together on the same plant modeled as a random factor. The effect of smoke particles on the larval, pupal and total mortality was analyzed by fitting Generalized Linear Mixed Models (GLMM) with a binomial distribution (surviving individuals were coded 1, dead ones 0) and a logit-link function^37^. The significance of the factors’ impact on survival was obtained by LRTs of full models tested against simplified models with the specific factors removed.

Results of experiments 1, 2 and 3 were analyzed separately because they were not conducted at the same time of the year and varied in rearing conditions. All analyzes were performed with R v. 3.2.4^38^ implemented in RStudio v. 1.0.136^39^. The following packages were used: nlme^40^, lsmeans^41^, multcomp^42^, MVN^43^ and lme4^44^.

## Results

### Smoke cages contained more smoke particles than control cages, but humidity and temperature were similar under both treatments

The multivariate analysis indicated that environmental conditions differed between the smoke and the control cages (MANOVA, Pillai’s Trace = 0.50, F_1,116_ = 38.12, p < 1 e^−12^). The average PM2.5 particle concentration was 117 µg m^−3^ in the smoke cage, and 50 µg m^−3^ in the control treatment, which represents a significant difference (ANOVA, F_1,116_ = 111.35; p < 1 e^−12^) (Fig. 1d). However, temperature and humidity were similar in both environments (ANOVA, temperature: F_1,116_ = 4.00 e^−3^, p = 0.95; humidity: F_1,116_ = 0.02, p = 0.88) (Fig. 1c,e). Because humidity and temperature were the same across all four cages (LRT: χ^2^_2_ = 0, p = 1), data from both cages of the same treatment in experiment 1 were pooled in subsequent analyses.

### Smoke significantly impacted the development of the caterpillars in both experiments 1 and 2

**Figure 2:**
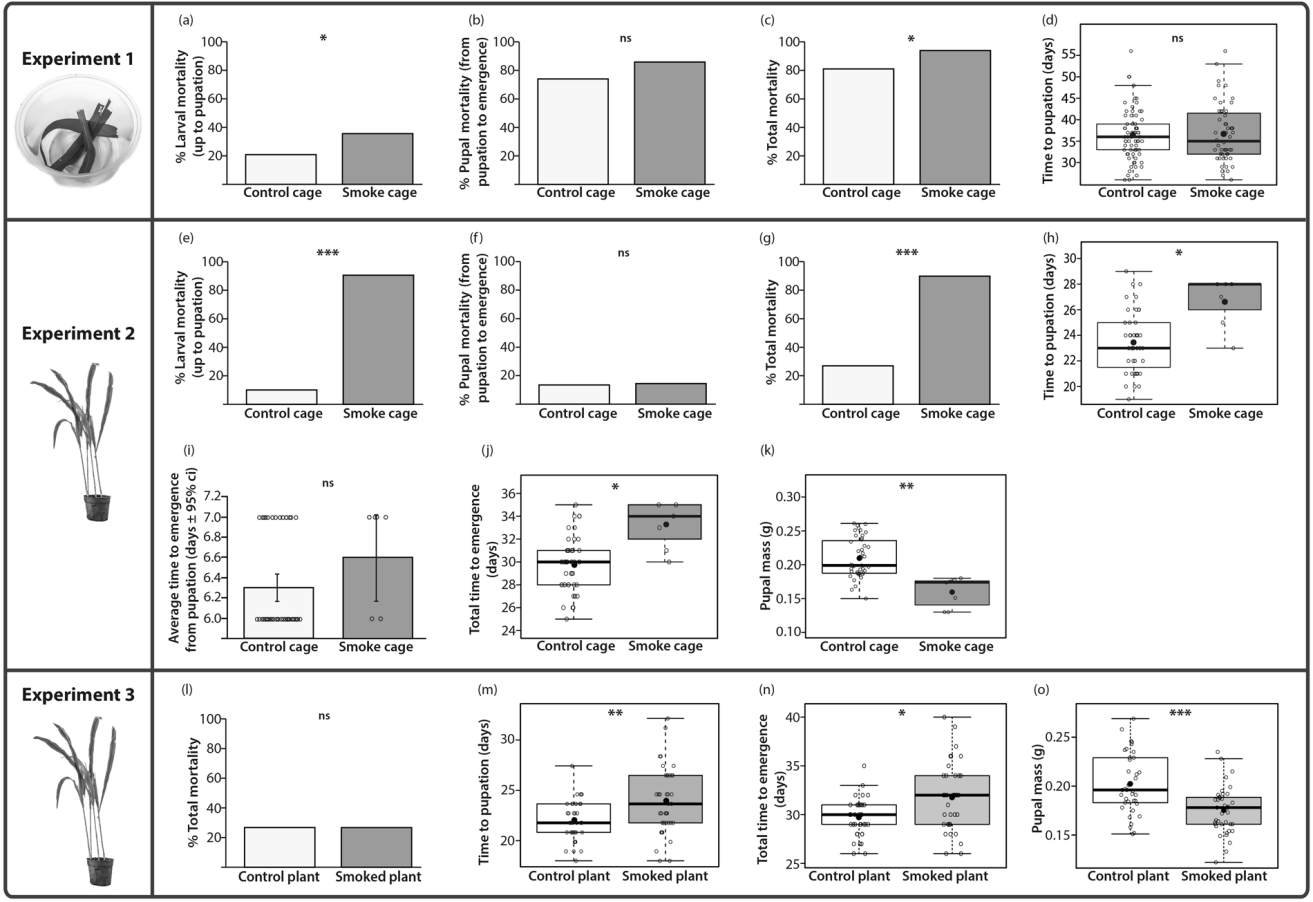
Smoke affected the development of caterpillars reared across the three experiments. In experiment 1, smoke significantly increased larval mortality (**a**), and total mortality (**c**), but didn’t impact pupal mortality (**b**), nor time to pupation (**d**). In experiment 2, smoke significantly increased larval mortality (**e**), total mortality (**g**) and time taken by the by larvae to reach the pupal stage (**h**). Smoke didn’t impact pupal mortality (**f**). Total development time from egg to butterfly emergence was longer in the smoke cage than in the control cage (**j**), but the time to emergence from pupation was similar in both cages (**i)**. Pupae from the smoke treatment were lighter that those from the control treatment (**k**). In experiment 3, smoked plants increased the time to pupation (**m**), the total time to emergence (**n**), and reduced the pupal mass (**o**). Total mortality was similar on both types of plants (**l**). In panels d, h, j, k, m, n and o, the higher and lower bars of the plot are the maximum and minimum values respectively, while the rectangle illustrates the first quartile, the median, and the third quartile (bottom to top). The black dot is the average and each open circle is a data point. Significant differences between treatments are represented by asterisks: *p < 0.05; **p < 0.01, ***p < 0.001, ns = non-significant.

In experiment 1, a significantly higher number of larvae died in the smoke treatment, resulting in fewer individuals reaching the pupal stage, compared to the control group (GLMM; χ^2^_1_ = 5.89, p = 0.02; Fig. 2a; Table 1). At the pupal stage there was a large mortality in both treatment groups, and only 25% of the control (17 individuals) and 12% of the smoke treated individuals (6 animals) emerged as butterflies (Table 1). There was no difference in pupal mortality between the two groups (GLMM; χ^2^_1_ = 3.14, p = 0.08; Fig. 2b), but total mortality from egg to butterfly was higher in the smoke treatment than in the control treatment (GLMM; χ^2^_1_ = 3.94, p = 0.05; Fig. 2c). However, smoke didn’t significantly impact time to pupation (LMM; F_1, 118_ = 0.05; p = 0.83) (Fig. 2d; Table 1), and the identity of the rearing pot did not impact any of the measured traits (for all dependent variables, LRT: χ^2^ = 0, p = 1).

**Table 1:** Summary of the impact of smoke on the development of caterpillars, in experiments 1, 2 and 3. n/a means ‘not-available’.

Experiment	Treatment	Starting number of larvae	Number of pupae	Percentage pupated	Average time to pupation in days (95% confidence interval)	Number of butterflies emerging from pupae	Percentage of emergence from pupae	Total percentage of emergence	Average time to emergence from pupae in days (95% confidence intervals)	Total average time to emergence in days (95% confidence intervals)
1	**Control cage**	88	69	78.41	36.45 (1.43)	17	24.65	19.32	n/a	n/a
	**Smoke cage**	80	51	63.75	36.71 (1.93)	6	11.77	7.50	n/a	n/a
2	**Control cage**	50	45	90	23.44 (0.74)	38	84.44	76	6.30 (0.14)	29.75 (0.74)
	**Smoke cage**	50	6	12	26.71 (1.83)	5	83.33	10	6.67 (0.54)	33.29 (1.90)
3	**Control plants**	50	37	74	22.29 (0.71)	37	100	74	7.43 (0.17)	29.73 (0.65)
	**Smoked plants**	50	39	78	24.33 (1.12)	37	94.87	74	7.35 (1.16)	31.78 (1.14)

In experiment 2, there was high larval mortality in the presence of smoke. Overall, 5 butterflies emerged from the 50 larvae reared in the smoke cage, whereas 38 adults emerged from the 50 individuals submitted to the control environment (Table 1). Smoke strongly reduced the probability of larvae surviving to the pupal stage and to butterfly emergence (GLMM; Larval mortality: χ^2^_1_ = 18.76, p = 1.48 e^−5^; Total mortality: χ^2^_1_ = 16.18, p = 5.77 e^−5^) (Fig. 1e,g). Smoke did not significantly impact pupal mortality (GLMM; χ^2^_1_ = 0.04, p = 0.84) (Fig. 1f) but larvae reared in the smoke cage took longer to reach the pupal stage compared to those reared in the control cage (LMM; F_1, 48_ = 11.72; p = 0.01) (Fig. 2h; Table 1). The average time to emergence from pupation was similar in both treatments, as all butterflies emerged after 6 or 7 days (LMM; F_1, 48_ = 3.16; p = 0.08) (Fig. 2i, Table 1), but larvae reared in the smoke cage took a significantly longer time to emerge as butterflies, compared to those reared in the control cage (LMM; F_1, 48_ = 13.54; p = 6.90 e^−4^) (Fig. 2j; Table 1). Pupae from the smoke cage were also significantly lighter than their control counterparts (LMM; F_1, 48_ = 18.83; p = 5.00 e^−3^) (Fig. 2k). Larval cohort didn’t impact any of the traits measured (LRT: χ^2^ = 0, p = 1).

### Smoked plants did not impact caterpillar survival but affected development time and pupal weight

Smoked plants didn’t affect larval and pupal survival. Larval mortality on smoked and control plants reached 22% and 26% respectively, and all the pupae from the control plant emerged, while 2 from the smoked plants died before emergence. Therefore, 74% of the individuals reached emergence in both treatments (GLMM; larval survival: χ^2^_1_ = 1.17 p = 0.68; pupal survival: χ^2^_1_ = 1.91, p = 0.17; total survival: χ^2^_1_ = 0, p = 1; Table 1, Fig. 2l). Despite similar survival across plant treatments, caterpillars reared on smoked plants took a bit longer to reach the pupal and the adult stages than caterpillars that grew on control plants (LMM; development time to pupae: F_1, 74_ = 8.73, p = 4.00 e^−3^; development time to adult: F_1, 72_ = 9.56, p = 0.02, Table 1, Fig. 2m,n). The pupae from individuals reared on smoked-plants were also smaller than the ones reared on control plants not exposed to the haze (LMM; F_1, 74_ = 18.22; p = 5.75 e^−5^, Table 1, Fig. 2o). Yet, adults reared on both types of plants took similar amount of days to emerge from the pupation step (LMM: F_1, 72_ = 0.50, p = 0.48, Table 1). Finally, the larval cohort didn’t affect any of the measured traits (LRT: χ^2^ = 0, p = 1).

### Histological examination of the trachea

**Figure 3:**
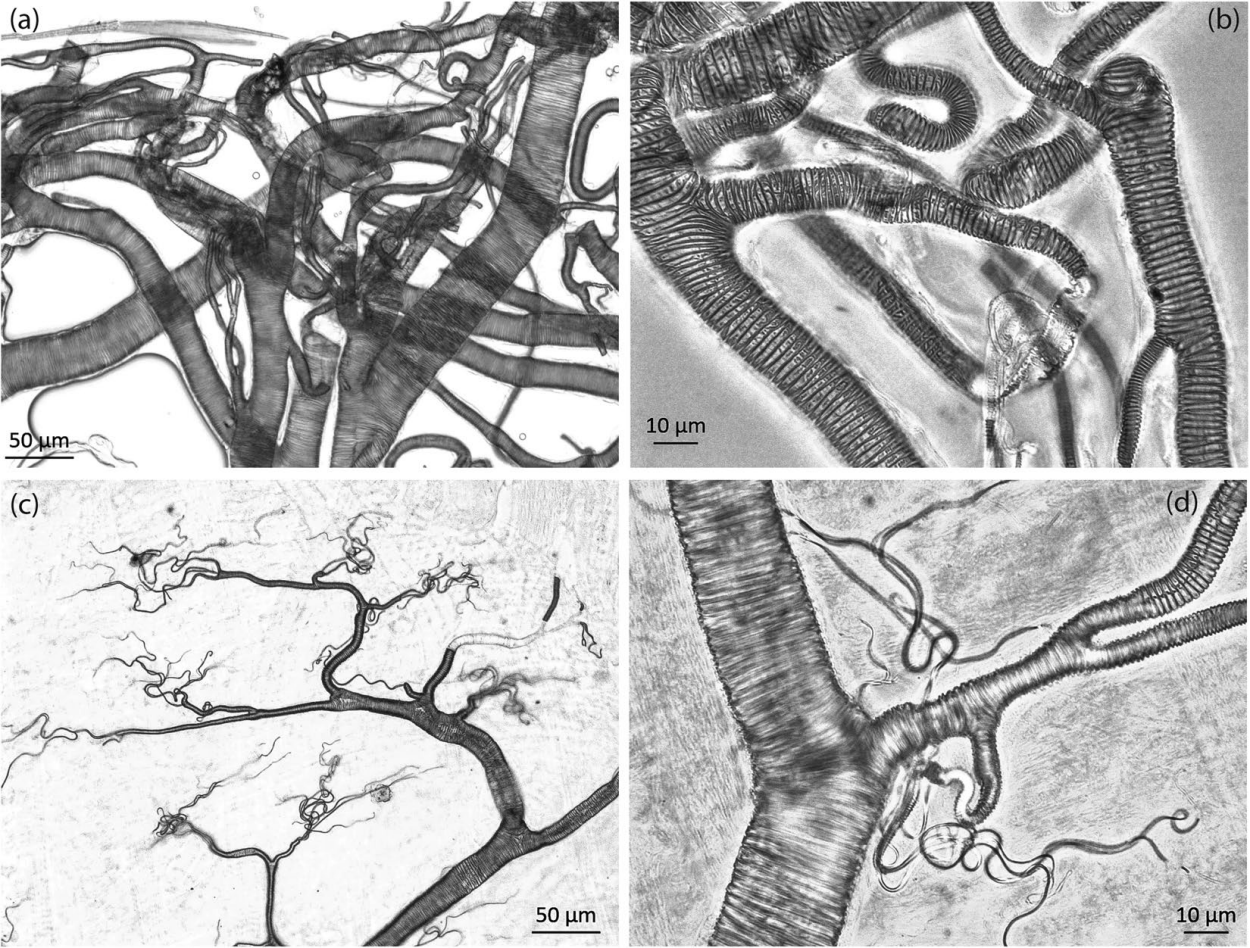
Tracheal samples dissected from caterpillars. (**a**,**b**) Trachea from caterpillars grown in the control cages and those grown in the smoke cages (**c**,**d**). Neither were obstructed by microparticles. Horizontal stripes seen on trachea, which are absent on the finest tracheoles with a diameter less than 1 µm, are helical thickenings called taenidia.

Microscopic examination of the trachea did not reveal any particle inside the respiratory tract of *B. anynana* larvae from the smoke or from the control group. Thoracic and abdominal sections of the trachea and tracheoles didn’t contain any visible particles that could be blocking the airways (Fig. 3).

### Scanning electron microscopy of spiracles

**Figure 4:**
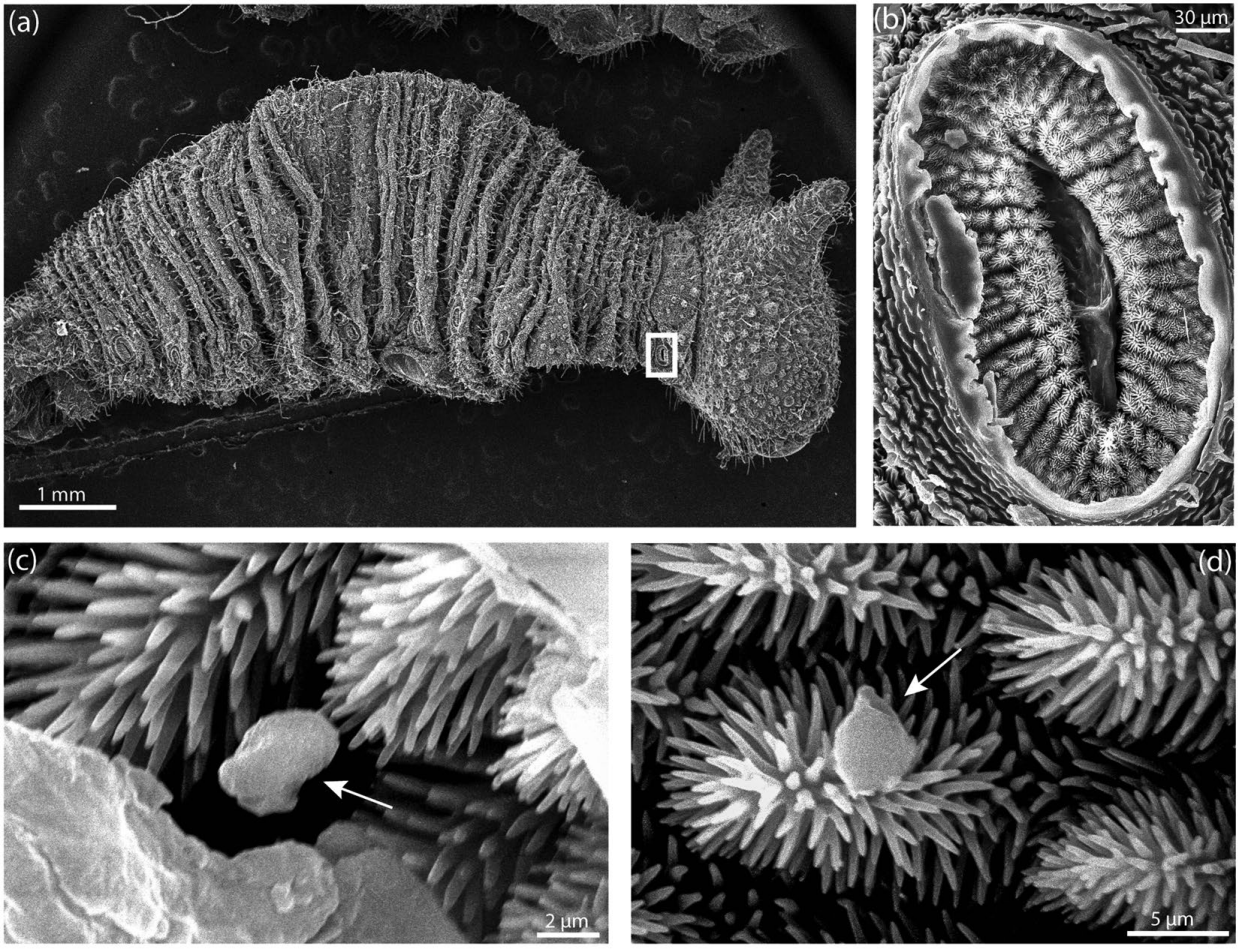
Scanning electron microscopy images of caterpillars and its thoracic spiracles. (**a**) A dried caterpillar reared in the smoke condition with a thoracic (air intake) spiracle highlighted by a white rectangle. (**b**) Enlarged view of thoracic spiracle. (**c**,**d**) Detail of trichomes covering the valves of the spiracles where a few micro-particles close to 2.5 µm are seen (arrow).

Examination of thoracic spiracles showed some particles trapped in the branched trichomes covering the spiracle’s entrance valves (Fig. 4). A few particles corresponding to the sizes of PM5 and PM2.5 were observed on the spiracle trichomes of one animal from the smoke treatment group only, but we could not quantify them since they were rare.

## Discussion

### Incense burning successfully generated PM2.5 at concentrations that simulated real haze episodes

The World Health Organization air quality guidelines recommend concentrations of PM2.5 to be kept below 25 µg m^−3^, over a 24 h period, in order to prevent damage to human health^45^. The Singapore National Environment Agency considers air quality unhealthy when PM2.5 rises above 55 µg m^−3^ over a 24 h period^46^. The high amount of PM2.5 in the smoke cages (117 µg m^−3^) suggested that the burning of coil incense was effective in producing unhealthy polluted air conditions, comparable to particular matter counts taken during the last haze events in Southeast Asia (SEA) countries. Haze episodes are recurrent in SEA, happening every 1 to 3 years since 1982^47,48^. In Singapore, the 2013 and 2015 episodes lasted around one and 2 months respectively, with [PM2.5] higher than 100 µg m^−3^ for 4 to 12 consecutive days, including 3 consecutive days when the concentration exceeded 230 µg m^−3^ in 2013^49–51^. In countries closer to the actual fires, the [PM2.5] remained dangerously high for long periods of time. For example, in 1997, the annual mean PM2.5 concentration reached 200 μg m^−3^ in Southeast Sumatra and Southern Borneo^12^. The September-October 2015 average [PM2.5] was above 300 μg m^−3^ in east Sumatra and south Kalimantan islands, Indonesia^52^. Human health and wildlife damages attributed to the haze are therefore expected to be extremely severe in these areas^12^.

Aside from differences in smoke particles measured across the two cage treatments, there were also likely differences in air composition across the two cages that were not quantified. Ambient temperature and humidity, however, were the same across all cages, so these environmental factors are unlikely to have contributed to differences in larval fitness observed across treatments.

### The smoke and the smoke-induced plant toxicity impaired the development of larvae

Caterpillars that grew subjected to continuous smoke, but not the ones that were fed with smoked-plants, suffered higher mortality in both experiments 1 and 2. The individuals reared in smoke in experiment 2 also had longer development time and lower pupal mass when compared to those that grew in clean air conditions. Individuals that were fed with smoked plants also had increased development time and reduced weight, although the effect sizes were smaller than in experiment 1. Therefore, it seems that the smoke alone induces caterpillar mortality, and that both the smoke and the plant toxicity induced by the smoke increased larval development time and reduced pupal weight, two important components of fitness. These results are in line with a meta-analysis which concluded that arthropods from areas with air pollution were generally smaller than those from control sites^53^. As smoke-treated larvae, and larvae fed with smoked plants have a reduced pupal mass, they likely resulted in smaller butterflies with reduced fecundity compared to the control-reared ones^54,55^. Since PM2.5 were absent from the tracheal system of the dead caterpillars reared in the smoke cage, we cannot conclude that the impaired development was caused by particles blocking the insect’s airways. We suggest, instead, that individuals, upon detection of poor air quality or air particles, closed their spiracle valves for longer time durations, decreasing O_2_ intake and reducing metabolism. Alternatively, toxic incense smoke components might have been responsible for the higher larval mortality, longer development time, and the lower pupal mass.

### The individuals reared in the smoke environment and on smoked plants may have suffered from food stress

Food deprivation was previously shown to lengthen the larval development time and produce smaller butterflies^56^, and may have originated from larvae having difficulty in locating the corn leaves, or from poor leaf quality. Pollutants such as nitrogen oxides, emitted in high quantities by burning incense and during forest fires^10,32^, enhance the formation of ground-level ozone by reacting with floral or foliar volatile compounds^57,58^. Ozone was shown to alter the plant volatiles that herbivores and pollinators use as cues to locate their food source, inducing them to search more and to forage less (e.g.^58,59^). In addition, ozone and sulfur components can both induce necrotic patches on the plant leaf surfaces and reduce the herbivore food quality^60–62^. Silkworm larvae, for instance, have non-uniform growth and delayed cocooning when they are on host plants exposed to SO_2_^63^. *Junonia coenia* caterpillars raised on foliage exposed to high-CO_2_ grew more slowly and experienced greater mortality, especially in early instars, than those raised on foliage exposed to low-CO_2_, probably due to the reduced foliar water and nitrogen concentrations of those plants^64^. Thus, the *B. anynana* caterpillars reared with incense may have had difficulties locating the corn leaves, may have breathed toxic compounds and ingested more toxic food than their control counterparts, leading to slower development and smaller pupal weight. In support of the first interpretation we also observed that larvae from the smoke treatment group had larger pieces of corn uneaten after each day of feeding, than larvae in the control treatment.

The consumption of smoked plants impacted larval development time and pupal weight less than the joint effect of the smoke and plant toxicity. Thus, in addition to food stress, the larvae from experiments 1 and 2 might have also suffered from chronic inflammation. As air exchange in insects occurs directly via diffusion through tracheoles to the surrounding tissues, acidic SO_2_ and NO_2_ from the smoke may directly cause tissue damage in the larvae, initiating an inflammation. Under certain stress factors, immune response signals can reduce larval growth rates and delay molting^65^. In mice, SO_2_, NO_2_, and PM2.5 induced inflammatory and endothelial dysfunction in the heart^66^.

Finally, the caterpillars from experiment 1 and 2 might have suffered from hypoxia, a deficiency in the amount of oxygen necessary for the tissue to work properly^67^. Although the proportion of gazes was not measured in our experiments, oxygen levels may have been lower in the smoke cages due to incense combustion. The air blown through the HEPA filter might have not been sufficient to renew the oxygen inside the smoke cage. Hypoxia often results in the reduction of insect survival, growth rate, body size, and reproduction, while inducing the damage of macromolecules and inflammation^67,68^. Insects can respond to hypoxia by decreasing their activities, such as feeding or moving^68^, increasing the risk of food stress. Insects can also compensate for a reduction of O_2_ in their environment by opening their spiracles and increasing abdominal pumping of air^68^, processes that also increase the intake of toxic gases. Testing these inter-linked hypotheses to identify the precise cause of increased mortality and development time in caterpillars growing in smoke environments remains open for future studies.

### Smoke did not impact pupal mortality

The similar pupal mortality observed in both treatments, and across the 3 experiments, suggests that smoke and the smoke-induced plant toxicity had little impact on pupal development. This is intriguing as there is high metabolic activity and oxygen demands during the late pupal stage especially^67^, and the air exchange increases^24^, including the intake of more toxic gas. In experiments 1 and 2, an insufficient sample size at the pupal stage caused by high mortality up to that point may have impaired the discovery of significant effects of smoke on pupal development. Alternatively, the low mortality at this stage might indicate that the effect of gases on food location and food quality are more important factors in reducing larval fitness than direct air toxicity, as pupae do not feed.

### Different types of food stress might have contributed to the different patterns of mortality and development time observed in experiments 1 and 2

Smoke-treated larvae fed in cups (experiment 1) suffered relatively less mortality compared to the ones fed on whole plants in experiment 2 (compare Fig. 2a with e). This difference cannot be explained by food toxicity since larval mortality in experiment 3 was low. However, locating the food might have been hard for the larvae in experiment 2, especially after the plants were replaced, because individuals were placed at the bottom of the pots rather than on top of the cut leaves (as in exp. 1). In addition, both caterpillar groups had longer larval development times in experiment 1 (~35 days) relative to experiment 2 (23 and 28 days) and 3 (~22 and 24 days). This increased development in experiment 1 might have led to overall smaller pupae and to higher pupal mortality. While we did not measure the weight of pupae in experiment 1, we observed that the time to pupation and pupal weight were inversely correlated in animals of experiment 2 and experiment 3. Feeding larvae in containers also led to an overall higher pupal mortality of both groups relative to the one measured in experiment 2 and 3 (compare Fig. 2b with f). This high mortality happened as most larvae entered into the pupation process, but failed to complete it, forming an unfinished pupa that would die after one day. The longer development time and higher pupal mortality in experiment 1 were likely due to food deprivation, as the corn amount allocated to the animals was rationed. Late larval stages of both treatment groups ate all the leaves allocated to them on a daily basis. This was not the case in experiment 2 and 3 where the animals were fed ad libitum on a live plant. These results are similar to earlier studies showing that starvation at the larval stage extended *B. anynana* larval development time, reduced the larval growth rate, increased the pupal mortality and decreased the butterfly body size, fecundity and reproductive investment^54,56^.

### The spiracles might be effective barriers against particulate matter (PM) and toxic gases

The valves of spiracles may function both as mechanical filters for PM as well as sensing organs that regulate spiracle closing and air intake. The absence of smoke particles in larvae trachea and the presence of particles trapped in the trichomes covering the spiracles suggest that these structures might be effective barriers against PM entering the larval body, as previously suggested^24^. This remains to be thoroughly tested. In addition, upon sensing particles, these trichomes might induce closure of the spiracle valve, preventing particles from entering the trachea. While we have no direct observations supporting this mechanism, mechanical disturbances are known to cause the closure of spiracles in cockroaches^69^. Similar muscles and innervation exist in lepidopteran spiracles^70^. Physiological studies also postulated that muscles that close the spiracles of Cecropia moth pupae are sensitive to O_2_ and CO_2_ levels thanks to receptors present near the spiracles^71,72^. Then, the potential CO_2_ increase inside the smoke cage could have induced longer spiracle closure than in the control cage in *B. anynana*.

## Conclusion

The present study is the first to highlight the deleterious impact of smoke on the development and survival of insects in a controlled laboratory experiment. Toxic chemicals, rather than particulate matter, together with smoke-induced plant toxicity, slow down development and lower pupal weight, while toxic chemicals in the smoke alone are the likely culprits in increasing larval mortality. This study takes a first step in understanding the impact of smoke on a population of butterflies. In natural environments, however, haze may affect multiple members of a complex food web, with less predictable outcomes. For example, in pollution-induced deteriorated areas, herbivore insects showed an increase in abundance because the density of their predators also decreased^53^. Additional studies, where the exact composition and gas levels are precisely controlled, for instance, are needed to further understand how the smoke haze affects the equilibrium of ecosystems.

## References

[1] Jaafar Z, Loh T-L (2014). Linking land, air and sea: potential impacts of biomass burning and the resultant haze on marine ecosystems of Southeast Asia. Global Change Biology.

[2] Lee JSH (2016). Toward clearer skies: Challenges in regulating transboundary haze in Southeast Asia. Environmental Science & Policy.

[3] Wang Y, Field RD, Roswintiarti O (2004). Trends in atmospheric haze induced by peat fires in Sumatra Island, Indonesia and El Niño phenomenon from 1973 to 2003. Geophysical Research Letters.

[4] Heil A, Goldammer J (2001). Smoke-haze pollution: a review of the 1997 episode in Southeast Asia. Regional Environmental Change.

[5] Lohman DJ, Bickford D, Sodhi NS (2007). The Burning Issue. Science.

[6] Forsyth T (2014). Public concerns about transboundary haze: A comparison of Indonesia, Singapore, and Malaysia. Global Environmental Change.

[7] Carrasco LR (2013). Silver lining of singapore’s haze. Science.

[8] Koplitz SN (2016). Public health impacts of the severe haze in Equatorial Asia in September–October 2015: demonstration of a new framework for informing fire management strategies to reduce downwind smoke exposure. Environmental Research Letters.

[9] Othman J, Sahani M, Mahmud M, Sheikh Ahmad MK (2014). Transboundary smoke haze pollution in Malaysia: Inpatient health impacts and economic valuation. Environmental Pollution.

[10] Radojevic Miroslav (2003). Chemistry of Forest Fires and Regional Haze with Emphasis on Southeast Asia. Air Quality.

[11] Davidson CI, Phalen RF, Solomon PA (2005). Airborne particulate matter and human health: A review. Aerosol Science and Technology.

[12] Marlier ME (2013). El Nino and health risks from landscape fire emissions in southeast Asia. Nature Climate Change.

[13] Sheldon TL, Sankaran C (2017). The impact of Indonesian forest fires on Singaporean pollution and health. American Economic review.

[14] Weisser, W. W. & Siemann, E. The various effects of insects on ecosystem functioning. In *Insects and Ecosystem Function. Ecological Studies (Analysis and Synthesis)* Vol. 173 (eds Weisser W. W. & Siemann E. (eds)) (Springer, 2008).

[15] Ollerton Jeff (2017). Pollinator Diversity: Distribution, Ecological Function, and Conservation. Annual Review of Ecology, Evolution, and Systematics.

[16] Willson, M. F. & Traveset, A. The ecology of seed dispersal. in *Seeds, 2rd Edition. The Ecology of Regeneration in* Plant Communities. (ed. Fenner, M.) 85–111 (CABI, 2000).

[17] Farwig N, Berens DG (2012). Imagine a world without seed dispersers: A review of threats, consequences and future directions. Basic and Applied Ecology.

[18] Bardgett RD, van der Putten WH (2014). Belowground biodiversity and ecosystem functioning. Nature.

[19] Miguel TB, Oliveira JMB, Ligeiro R, Juen L (2017). Odonata (Insecta) as a tool for the biomonitoring of environmental quality. Ecological Indicators.

[20] Ghannem S, Touaylia S, Boumaiza M (2018). Beetles (Insecta: Coleoptera) as bioindicators of the assessment of environmental pollution. Human and Ecological Risk Assessment: An International Journal.

[21] Hill KJ, Hamer K, Lace L, Banham MT (1995). W. Effects of selective logging on tropical forest butterflies on Buru, Indonesia. Journal of Applied Ecology.

[22] Cleary DFR, Grill A (2004). Butterfly response to severe ENSO-induced forest fires in Borneo. Ecological Entomology.

[23] Chown, S. L. & Nicolson, S. *Insect physiological ecology: mechanisms and patterns*. (Oxford University Press, 2004).

[24] Nikam, T. B. & Khole, V. V. *Insect spiracular systems*. (Ellis Horwood Limited, 1989).

[25] Hartung DK, Kirkton SD, Harrison JF (2004). Ontogeny of tracheal system structure: A light and electron-microscopy study of the metathoracic femur of the American locust, *Schistocerca americana*. Journal of Morphology.

[26] Socha JJ, Förster TD, Greenlee KJ (2010). Issues of convection in insect respiration: Insights from synchrotron X-ray imaging and beyond. Respiratory Physiology & Neurobiology.

[27] Westneat MW (2003). Tracheal respiration in insects visualized with synchrotron x-ray imaging. Science.

[28] Greenlee KJ (2013). Hypoxia-induced compression in the tracheal system of the tobacco hornworm caterpillar. Manduca sexta. The Journal of Experimental Biology.

[29] Pendar H, Kenny MC, Socha JJ (2015). Tracheal compression in pupae of the beetle *Zophobas morio*. Biology letters.

[30] Wasserthal Lutz Thilo, Cloetens Peter, Fink Rainer H., Wasserthal Lennard Knut (2018). X-ray computed tomography study of the flight-adapted tracheal system in the blowflyCalliphora vicina, analysing the ventilation mechanism and flow-directing valves. The Journal of Experimental Biology.

[31] Gerolt P (1983). Insecticides: their route of entry, mechanism of transport and mode of action. Biological Reviews.

[32] Jetter JJ, Guo Z, McBrian JA, Flynn MR (2002). Characterization of emissions from burning incense. Science of The Total Environment.

[33] Lee S-C, Wang B (2004). Characteristics of emissions of air pollutants from burning of incense in a large environmental chamber. Atmospheric Environment.

[34] Siao WS, Balasubramanian R, Joshi UM (2007). Physical characteristics of nanoparticles emitted from incense smoke. Science and Technology of Advanced Materials.

[35] Zvereva EL, Kozlov MV (2006). Consequences of simultaneous elevation of carbon dioxide and temperature for plant–herbivore interactions: a metaanalysis. Global Change Biology.

[36] Millar RB, Anderson MJ (2004). Remedies for pseudoreplication. Fisheries Research.

[37] Zuur, A. F., Ieno, E. N., Walker, N. J., Saveliev, A. A. & Smith, G. M. Mixed Effects Modelling for Nested Data. In *Mixed effects models and extensions in ecology with R* 101–142 (Springer New York, 2009).

[38] R: A language and environment for statistical computing. (R Foundation for Statistical Computing, Vienna, Austria, 2016).

[39] RStudio: integrated development for R. (RStudio, Inc., Boston, MA, 2015).

[40] nlme: linear and nonlinear mixed effects models v. R package version 3.1–131 (2017).

[41] Lenth RV (2016). Least-Squares Means: The R Package lsmeans. Journal of Statistical Software.

[42] Hothorn T, Bretz F, Westfall P (2008). Simultaneous inference in general parametric models. Biometrical Journal.

[43] Korkmaz S, Goksuluk D, Zararsiz G (2014). MVN: An R Package for Assessing Multivariate Normality. The R Journal.

[44] Bates D, Maechler M, Bolker B, Walker S (2015). Fitting linear mixed-effects models using lme4. Journal of Statistical Software.

[45] World Health Organization. *Ambient air pollution: A global assessment of exposure and burden of disease*. (World Health Organization, 2016).

[46] National Environment Agency of Singapore. *Computation of the Pollutant Standards Index (PSI)*, https://www.haze.gov.sg/docs/default-source/faq/computation-of-the-pollutant-standards-index-%28psi%29.pdf (2014).

[47] See SW, Balasubramanian R, Wang W (2006). A study of the physical, chemical, and optical properties of ambient aerosol particles in Southeast Asia during hazy and nonhazy days. Journal of Geophysical Research D: Atmospheres.

[48] Field, R. D. & Shen, S. S. P. Predictability of carbon emissions from biomass burning in Indonesia from 1997 to 2006. *Journal of Geophysical Research: Biogeosciences* **113** (2008).

[49] Betha R, Behera SN, Balasubramanian R (2014). 2013 Southeast Asian Smoke Haze: Fractionation of Particulate-Bound Elements and Associated Health Risk. Environmental Science & Technology.

[50] Gaveau DLA (2014). Major atmospheric emissions from peat fires in Southeast Asia during non-drought years: evidence from the 2013 Sumatran fires. Scientific Reports.

[51] Kusumaningtyas S, Aldrian E (2016). Impact of the June 2013 Riau province Sumatera smoke haze event on regional air pollution. Environmental Research Letters.

[52] Crippa P (2016). Population exposure to hazardous air quality due to the 2015 fires in Equatorial Asia. Scientific Reports.

[53] Zvereva EL, Kozlov MV (2010). Responses of terrestrial arthropods to air pollution: a meta-analysis. Environmental Science and Pollution Research.

[54] Bauerfeind SS, Fischer K (2005). Effects of food stress and density in different life stages on reproduction in a butterfly. Oikos.

[55] Rosa E, Saastamoinen M (2017). Sex-dependent effects of larval food stress on adult performance under semi-natural conditions: only a matter of size?. Oecologia.

[56] Saastamoinen M, van der Sterren D, Vastenhout N, Zwaan BJ, Brakefield PM (2010). Predictive adaptive responses: condition‐dependent impact of adult nutrition and flight in the tropical butterfly *Bicyclus anynana*. The American Naturalist.

[57] Pierce T (1998). Influence of increased isoprene emissions on regional ozone modeling. Journal of Geophysical Research.

[58] McFrederick QS, Kathilankal JC, Fuentes JD (2008). Air pollution modifies floral scent trails. Atmospheric Environment.

[59] Fuentes J, Roulston TH, Zenker J (2013). Ozone impedes the ability of a herbivore to find its host. Environmental Research Letters.

[60] Novak K (2007). Ozone air pollution effects on tree-ring growth, δ13C, visible foliar injury and leaf gas exchange in three ozone-sensitive woody plant species. Tree Physiology.

[61] Wilkinson S, Mills G, Illidge R, Davies WJ (2012). How is ozone pollution reducing our food supply?. Journal of Experimental Botany.

[62] Rhimi N (2016). Morpho-anatomical and physiological changes in grapevine leaves exposed to amospheric fluoride and sulfur dioxide pollution. Applied Ecology and Environmental Research.

[63] Alstad D, Edmunds G, Weinstein L (1982). Effects of air pollutants on insect populations. Annual Review of Entomology.

[64] Fajer ED, Bowers MD, Bazzaz FA (1989). The Effects of Enriched Carbon Dioxide Atmospheres on Plant—Insect Herbivore Interactions. Science.

[65] Lavine MD, Strand MR (2002). Insect hemocytes and their role in immunity. Insect Biochemistry and Molecular Biology.

[66] Zhang Y, Ji X, Ku T, Sang N (2016). Inflammatory response and endothelial dysfunction in the hearts of mice co-exposed to SO2, NO2, and PM2.5. Environmental Toxicology.

[67] Hoback WW, Stanley DW (2001). Insects in hypoxia. Journal of Insect Physiology.

[68] Harrison JF, Greenlee KJ, Verberk WCEP (2018). Functional hypoxia in insects: definition, assessment, and consequences for physiology, ecology, and evolution. Annual Review of Entomology.

[69] Case JF (1957). The median nerves and cockroach spiracular function. Journal of Insect Physiology.

[70] Schmitz A, Wasserthal LT (1999). Comparative morphology of the spiracles of the Papilionidae, Sphingidae, and Saturniidae (Insecta: Lepidoptera). International Journal of Insect Morphology and Embryology.

[71] Burkett BN, Schneiderman HA (1974). Roles of oxygen and carbon dioxide in the control of spiracular function in Cecropia pupae. Biology Bulletin.

[72] Burkett BN, Schneiderman HA (1974). Discontinuous respiration in insects at low temperatures: intratracheal pressure changes and spiracular valve behavior. Biology Bulletin.

